# ENGAGING WITH AGING: OLDER ADULTS’ ADAPTATIONS TO URINARY CONCERNS

**DOI:** 10.1093/geroni/igad104.3303

**Published:** 2023-12-21

**Authors:** Kuan-Ching Wu, Shaoqing Ge, Shao-Yun Chien, Jessica Jin, Suah Park, Basia Belza

**Affiliations:** University of Washington, Seattle, Washington, United States; University of Washington, Seattle, Washington, United States; University of Washington, Seattle, Washington, United States; University of Washington, Seattle, Washington, United States; University of Washington, Seattle, Washington, United States; University of Washington, Seattle, Washington, United States

## Abstract

Urinary concerns (UC) increase with age impacting health and quality of life. The aims of this study were to describe: 1) UC as an age-related change (ARC); 2) the challenges of UC; 3) adaptation strategies used to manage UC; and 4) the value of engaging with aging (EWA) as a framework to promote self-management of UC. Data was used from semi-structured interviews with 29 older adults (mean age 77 years). An iterative coding process was used. A codebook was developed based on a-priori themes derived from the EWA framework, our previous publication, and a line-by-line coding of one of the transcripts. As the analysis progressed, additional codes emerged, enriching the codebook. Six themes emerged: 1) the participants’ experiences of UC; 2) responses to UC, 3) adaptation and management strategies; 4) knowledge and understanding of UC; 5) available capacities and resources; and 6) the impact of the COVID-19 pandemic on UC. Participants tended to address their UC by adjusting routines, medication schedules, or diet patterns. They tried to secure restroom locations or use tools or reminders to resolve their UC. COVID-19 led to increased inconvenience for older adults to engage in outdoor activities due to the closure of public restrooms. Through a comprehensive qualitative study and in-depth analysis of older adults’ UC and adaptations, these participants developed personalized adjustments to address their needs and abilities to their UC. These findings offer insights into the individual aging experience, which will further enhance our understanding and advancement of person-centered care.

